# Large area Germanium Tin nanometer optical film coatings on highly flexible aluminum substrates

**DOI:** 10.1038/srep34030

**Published:** 2016-09-26

**Authors:** Lichuan Jin, Dainan Zhang, Huaiwu Zhang, Jue Fang, Yulong Liao, Tingchuan Zhou, Cheng Liu, Zhiyong Zhong, Vincent G. Harris

**Affiliations:** 1 State Key Laboratory of Electronic Thin Films and Integrated Devices, University of Electronic Science and Technology of China, Chengdu 610054, People’s Republic of China; 2 Department of Electrical and Computer Engineering, University of Delaware, Newark, Delaware 19716, United States; 3 Department of Electrical and Computer Engineering, Northeastern University, Boston, Massachusetts 02115, United States

## Abstract

Germanium Tin (GeSn) films have drawn great interest for their visible and near-infrared optoelectronics properties. Here, we demonstrate large area Germanium Tin nanometer thin films grown on highly flexible aluminum foil substrates using low-temperature molecular beam epitaxy (MBE). Ultra-thin (10–180 nm) GeSn film-coated aluminum foils display a wide color spectra with an absorption wavelength ranging from 400–1800 nm due to its strong optical interference effect. The light absorption ratio for nanometer GeSn/Al foil heterostructures can be enhanced up to 85%. Moreover, the structure exhibits excellent mechanical flexibility and can be cut or bent into many shapes, which facilitates a wide range of flexible photonics. Micro-Raman studies reveal a large tensile strain change with GeSn thickness, which arises from lattice deformations. In particular, nano-sized Sn-enriched GeSn dots appeared in the GeSn coatings that had a thickness greater than 50 nm, which induced an additional light absorption depression around 13.89 μm wavelength. These findings are promising for practical flexible photovoltaic and photodetector applications ranging from the visible to near-infrared wavelengths.

Flexible photonics have been developed to meet the demands for wearable electronic devices among other applications. To meet the needs of high efficiency photovoltaic ultrathin silicon solar cells, new forms of Si (nano/microwires, surface patterning and hybrid heterojunctions) films have emerged to enhance the light absorption^1^,^2^,^3^,^4^. Wang *et al*. in 2013 fabricated 10.7 to 1.6 μm flexible single crystal silicon wafers by KOH solution etching^5^. They showed that light absorption significantly depended on the silicon thickness, which varied from 552 nm to 790 nm. Li Zhu *et al*. in 2015 experimentally demonstrated brilliant colors tunable from green to red on a silicon film embedded on a flexible membrane^6^. The results were attractive for flexible optical applications. However, Si is an indirect band gap semiconductor with low carrier mobility, which is not suitable for high efficiency photovoltaic applications.

Germanium Tin (GeSn) is one of the most important tunable band gap material systems for visible and near/mid-infrared optoelectronic applications^7^,^8^,^9^,^10^. GeSn exhibits a direct band gap for Sn concentrations above 6.5 atomic percent, which is very promising for on-chip integrated optical detectors and modulators^11^. Additionally, the lattice constant of GeSn can be tuned by varying the Sn composition to allow for heteroepitial growth of multifunctitonal heterostructures^12^,^13^. Since the solubility limit of Sn in Ge is relatively low, i.e., ~1%, low temperature molecular beam epitaxy (MBE) and chemical vapor deposition (CVD) techniques have been developed to realize the growth of GeSn films with high Sn composition^14^,^15^. Recently, K. Toko deposited polycrystalline GeSn with Sn composition exceeding 25% on flexible plastic substrates at a extremely low temperature of 70 °C 16. In order to achieve ultrathin and highly absorbing optical films, F. Capasso *et al*. proposed to deposit a few tens of nanometers thick high dielectric Ge films on a metallic Au layer^17^. The Ge films require a minimum amount of absorbing material that can be as thin as 5–20 nm for visible light (400–850 nm). The findings open a new route for the ultrathin photodetector and solar cells applications.

In this work, we fabricated nanometer thick GeSn optical thin films on highly flexible aluminum (Al) foil substrates using low temperature MBE technique. The GeSn thickness dependent of optical absorption cutoff wavelength was realized. Color spectrum shows that its color gradually changes from bright yellow, to dark blue due to insufficient light absorption. The obtained absorption cutoff wavelength can be tuned from 400 nm to 1800 nm. However, the films thickness is much smaller than a quarter-wave thickness due to strong light interference. The size of the ultrathin GeSn optical coating can be larger than 4 inch diameter, which is very promising for practical flexible photovoltaic and photodetector applications ranging from visible to near-infrared wavelengths.

## Results and Discussions

**Figure 1:**
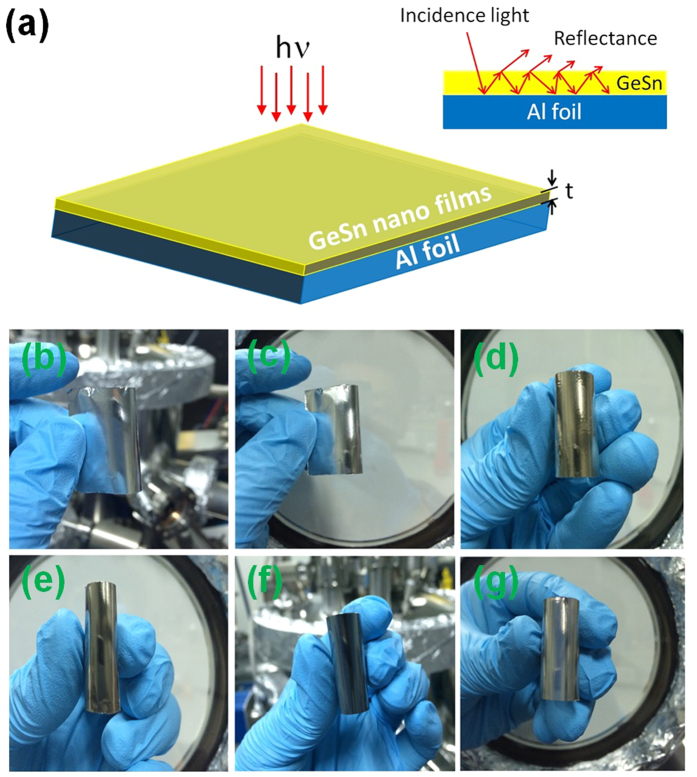
(**a**) Schematic of the GeSn nano films with thickness of *t* coating on Al foils. (**b–g**) Widely color spectra of the nanometer GeSn coating on Al foils, *t* = 0, 20, 40, 60, 100 and 180 nm (Photos are taken under illumination from conventional fluorescent ceiling lights).

Figure 1(a) shows the schematic of the GeSn film with thickness *t* on flexible Al foil. Inset graph describes the behavior of light (red arrow) incident from air into the highly absorptive GeSn nanometer thin film as an optical coatings, which was deposited on a metallic Al foil substrate. We assume that there is no transmission through the Al foil substrate, the absorption of the structure can be written as A = 1 − R, where A is light absorption and R is the light reflection. For a metal Al foil substrate in the perfect electric conductor limit, its’ complex refractive index 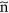
_Al_ = *n*_Al_ + *ik*_Al_, *n*_Al _→ ∞ and *k*_Al_ → ∞. The incident light is completely reflected at the Al-GeSn interface with a phase shift of π, which makes the GeSn thickness much lower than the wavelength with *h* ≈ *λ*/4*n*_GeSn_ (*n*_GeSn_ is the refractive index of the GeSn). Figure 1(b–g) presents a photograph of samples of Al foil coated GeSn from 0 to 180 nm in thickness, which creates a spectrum of colors including silver, golden, dark blue and light blue. Although the surface of the Al foil substrates are unpolished, the various colors still clearly appear. It should be mentioned that the Al foil is polycrystalline with a thickness of ~200 μm. The grain distribution changes from 102 nm to 238 nm, which has been checked using an atomic force microscopy. The findings agree well with a previous report^18^. The wide optical absorption band comes from the remarkable reflectivity change of the aluminum foil by coating it with nanometer thick GeSn films. GeSn was selected because it is highly absorbing at visible/near-infrared wavelengths. Moreover, its indirect band gap can be tuned to a direct band gap with the application of tensile strain, which is very important for its photovoltaic and photodetector applications. The samples exhibit excellent mechanical flexibility and do not crack even after repeated bending. It can be cut into any shapes by shears, which facilitate flexible photonics fabrication.

**Figure 2:**
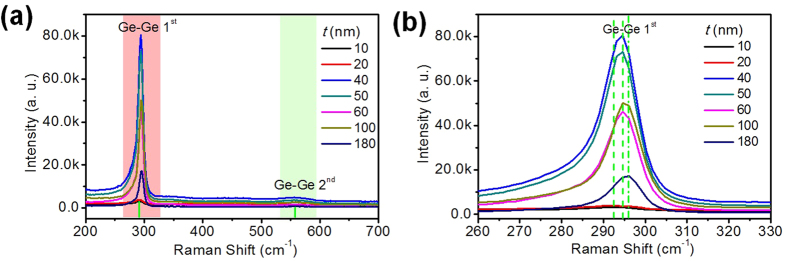
(**a**) Micro-Raman spectroscopy measurement for the nanometer GeSn thin films coating on Al foil with different *t*. (**b**) Enlarged Ge-Ge peak shift as function of GeSn thickness.

Room temperature micro-Raman spectroscopy for GeSn nanometer thin film coatings on Al foil with different thicknesses are shown in Fig. 2(a). A HeNe 532 nm laser was used as an excitation source. The exact wavenumber position of the peaks reflects the influence of chemical composition and strain. All Raman spectra present a strong Ge-Ge 1^st^ peak located at ~292 cm^−1^. While, Ge-Ge 2^nd^ peak locates at ~544 cm^−1^. It compares well with previous results of GeSn epitaxial growth on Si and SiO_2_^19^,^20^,^21^. The typical Ge-Sn peak always displays at lower wavenumber 250-260 cm^−1^. Actually, the shoulder on the left side of the Ge-Ge 1^st^ peak is due to the Ge-Sn peak at ~285 cm^−1^ 22. The typical Ge-Al peak is at ~370 cm^−1^ 23, however, in this work the Al implanted GeSn can be excluded from the Raman spectra.

Figure 2(b) shows the enlarged Ge-Ge 1^st^ peak, from which a clear peak shift towards higher wavenumber with increasing GeSn thickness is observed. Comparing the Ge-Ge 1^st^ peak at 292.7 cm^−1^ for 10 nm GeSn, a wavenumber shift of 3.1 cm^−1^ was achieved for 180 nm GeSn coated Al foils. It indicates that lattice strain changes much with changing GeSn coating thicknesses. The change in residual strain in epitaxial films was proven arising from lattice deformation^24^. We assume that the lattice deformation becomes larger for thicker GeSn films grown on the Al foil substrates. Ishikawa *et al*. demonstrated both the growth temperature and the thickness might affect the strain in the GeSi system^25^.

**Figure 3:**
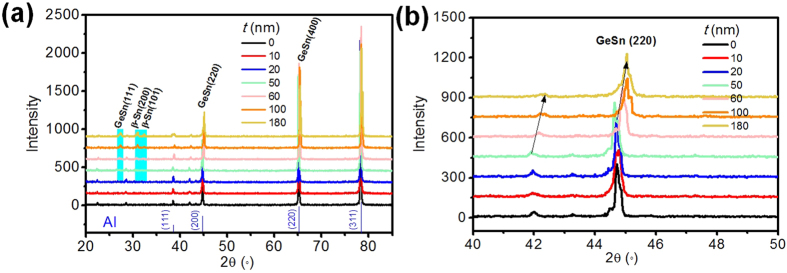
(**a**) XRD patterns of GeSn nanometer coating on Al foil. (**b**) Enlarged XRD patterns with 2θ ranging from 40° to 50°.

In order to describe the lattice deformation, we study the microstructure of the GeSn nanometer coatings on Al foil using XRD. The XRD patterns are shown in Fig. 3(a), most visible are the Al peaks from the substrate. With GeSn thickness *t* increasing up to 50 nm, GeSn (111) phase and GeSn (220) phase gradually appear. β-Sn (200) and (101) phases also appear corresponding to samples of high relative thickness. Raman analysis proved that the lattice strain changes considerably for different thicknesses of GeSn films. Here, as shown in Fig. 3(b), a large GeSn (220) peak shift is observed in the enlarged XRD patterns. The higher diffraction angle indicates a change to smaller lattice constants, which means the lattice constant of GeSn decreases for *t* larger than 50 nm. It has been shown that the epitaxial breakdown would change the surface morphology from a 2D growth mode to a 3D growth mode with large islands^26^. It is conjectured that Sn-rich precipitates will form on the GeSn thin films with *t* larger than 50 nm. The composition of the GeSn film changes with epitaxial breakdown, which can explain the decrease of GeSn lattice constant and the concomitant appearance of the β-Sn phase.

**Figure 4:**
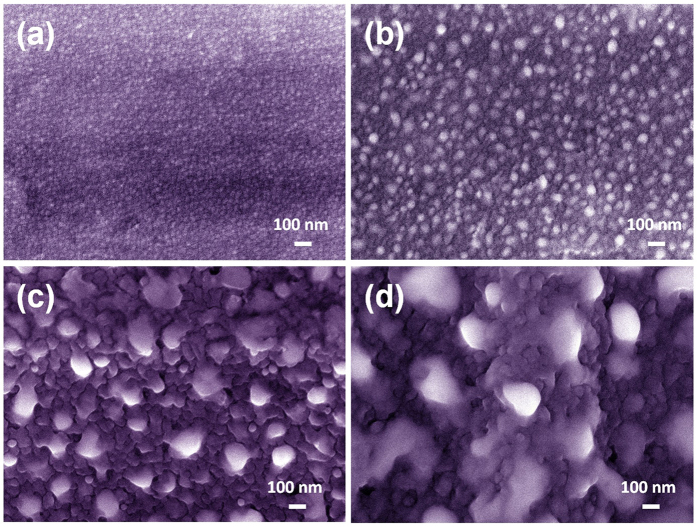
High resolution SEM images of GeSn thin film coating on Al foils (**a**) t = 20 nm (**b**) t = 50 nm (**c**) t = 100 nm (**d**) t = 180 nm.

**Figure 5:**
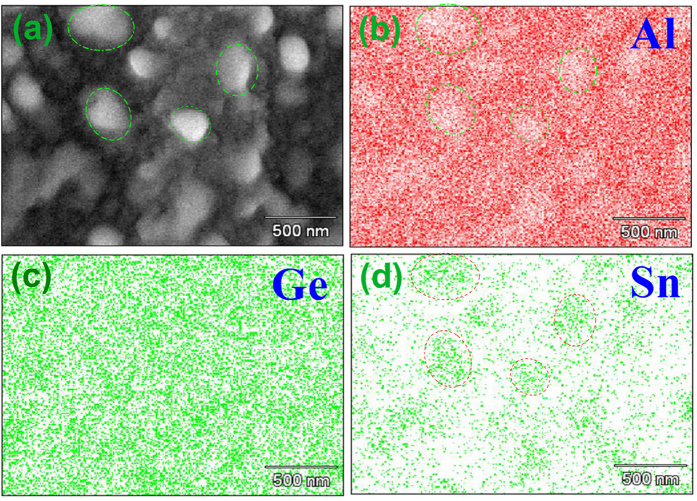
EDS of GeSn films with a thickness of 180 nm grown on Al foil (**a**) SEM image of GeSn with a thickness 180 nm grown on Al foil. (**b**) Al element mapping. (**c**) Ge element mapping. (**d**) Sn element mapping.

To better understand the nanoscale structure of GeSn thin film coated Al foils, high-resolution SEM images were measured. Figure 4(a–d) show the SEM images of GeSn thin films grown on Al foil with *t* = 20, 50, 100 and 180 nm, respectively. The GeSn thin film coatings present a smooth surface even grown upon normal Al foil substrate that were largely untreated from their commercial state. However, it is clear that nano-sized islands gradually atop the GeSn coatings that are thicker than 50 nm, as shown in Fig. 4(b–d). The XRD results suggest there probably exists Sn-rich islands. Here, we indeed observe these nano islands from the SEM images. To identify the elemental makeup of these nano-sized islands, we performed elemental mapping of GeSn films with a thickness of 180 nm grown on Al foil. As shown in Fig. 5(b), the Al element distributes uniformly with several light areas (in circled dash lines). Comparing this with the Sn element mapping as illustrated in Fig. 5(d), the nano islands appear Sn-rich as GeSn blotches. The results indicate an epitaxial breakdown exists in MBE grown GeSn thin films on Al foil substrate for thicknesses greater than 50 nm, which changes the surface morphology from a 2D growth mode to a 3D growth mode with relatively larger nm islands.

**Figure 6:**
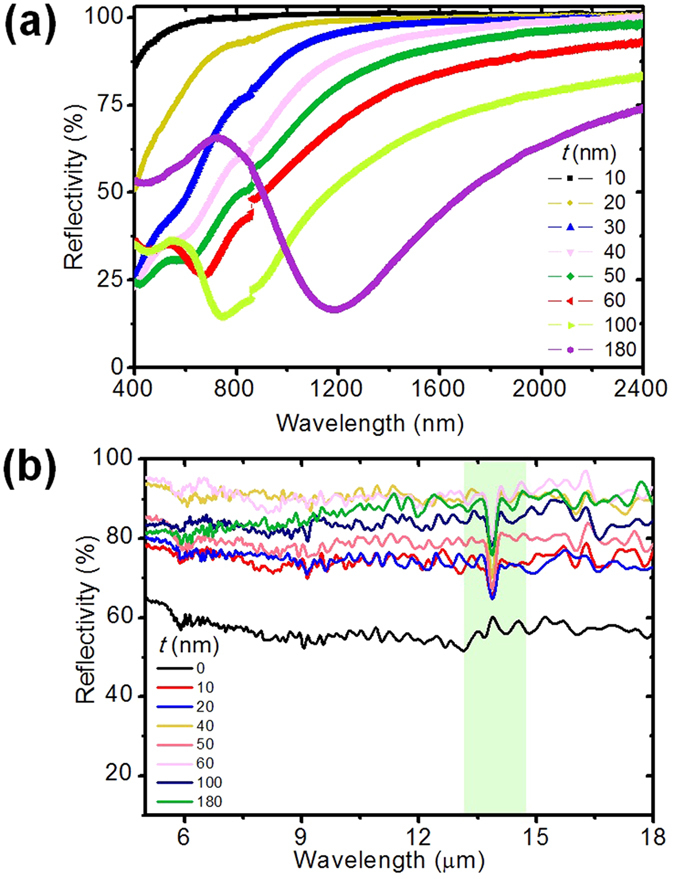
(**a**) Refection spectra (from 400 to 2400 nm in wavelength) of Al foil coated with 10, 20, 30, 40, 50, 60, 100 and 180 nm of GeSn thin films. (**b**) Refection spectra (from 5 to 18 μm in wavelength) of Al foil coated with various thickness of GeSn thin films.

The visible/infrared reflection spectra were obtained using a Vis/NIR spectrophotometer (Lambda750). The incident light was unpolarized with an incident angle of ~5° with respect to the Al foil’s normal. An integrating sphere is used to collect the light back scattered in all directions. The reflectivity of a bare Al foil was employed as a reference. Figure 6(a) shows the measured reflection spectra of Al foil coated with various thicknesses of GeSn thin films (where *t* = 10, 20, 30, 40, 50, 60, 100, and 180 nm) over a wavelength range of 400–2400 nm. It is noticed that the change in reflectivity for different *t* samples is remarkable. With an increase of GeSn coating thickness, the response wavelength (the light reflectivity <50%) can be tailored for a range of wavelengths from 400 nm to 1800 nm. From the discussion above, without considering the Al transmission, the optical absorption of GeSn films can be obtained as A = 1 − R. The maximum light absorption ratio is as high as 85%, while a dip in reflectance is around 15% at *t* = 100 nm. The reflectivity spectra have the same shape and tend to a red shift with increasing GeSn coating thickness on Al foil substrate. The results agree well with the evolution of the GeSn/Al foil color as presented in Fig. 1(b). The enhanced light absorption comes from the light interference effect in GeSn/Al foil structure, which has great potential to be employed in highly efficient photovoltaic and photodetector applications. However, the epitaxial breakdown for thick GeSn films will reduce the efficiency of photovoltaics due to the Sn segregation induced poor layer quality. Especially, the epitaxial breakdown will cause a large surface roughness and a crystalline structure degradation. In order to suppress the Sn segregation, a minimum growth rate is preferred^27^. Figure 6(b) shows the reflection spectra in the range of 5–18 μm measured by FTIR. The reflection spectrum of bare Al foil has a uniform reflectance of ~60% (as shown as the black curve). The reflectance is boosted ~20 to 35% with coatings of GeSn nanometer films. In addition, there is an additional absorption dip around 13.89 μm. The relation between the wavelength of absorption light and the energy band gap *E*_g_ is given by *λ*_abs_ = 1.24/*E*_g_ 25. So, *E*_g_ is extracted as 0.089 eV. This agrees well with the band gap of Tin (~0.08 eV).

## Conclusions

In summary, nanometer thick GeSn thin film coatings were deposited on highly flexible aluminum foil substrates using a low-temperature MBE approach. The optical absorption wavelength can be greatly tuned from 400 nm to 1800 nm with a film thickness much smaller than the quarter-wavelength thickness due to strong light interference effects. Various colors (silver, golden, dark blue and light blue) were obtained for the GeSn coated foils. Raman and XRD studies indicate that lattice strain changes considerably with different GeSn coating thicknesses. Nano-sized Sn-enriched GeSn islands gradually appeared on the GeSn coatings of thickness greater than 50 nm, which induced an additional absorption depression around 13.89 μm wavelength. The light absorption ratio in the nanometer GeSn/Al foil structures can be enhanced up to 85%, which has great potential to be used in highly efficient photovoltaic applications. Most importantly, the samples exhibit excellent mechanical flexibility with no apparent effects experienced by repeated bending, which facilitates their use in flexible photonics device technology.

## Methods

The GeSn films of the present study were deposited on Al foils using a MBE system with a base pressure lower than 2 × 10^−10^ torr. The growth temperature was fixed at 250 °C. The crucible temperature for the Ge source was 1200 °C, while the crucible temperature for Sn source was 1000 °C. The thickness of the GeSn films were controlled by controlling the duration of deposition and knowing the growth rate was 1 nm/min.

Aluminum block in 99.99% purity has been squeezed to reduce its thickness to less than 200 μm with several passes through rolling mills. Lubrication has been used to facilitate the rolling process and smoothen the foil surface. In the final pass, two sheets are packed together and rolled simultaneously, which creates bright sides in contact with rolls and matte sides in contact with the other sheet. The aluminum foil is appropriately annealed during the rolling process to maintain its workability.

X-ray photoelectron spectroscopy (XPS) was carried out to quantitatively analyze the elemental composition and chemical ionic state of the elements. Micro-Raman analysis revealed evidence of interfacial strain for the GeSn film. The microstructure of GeSn coatings was examined using X-ray diffraction (XRD). High resolution scanning electron microscopy (HRSEM) was used to investigate the surface morphology of the films. Elemental mapping has been carried out using energy dispersive x-ray spectroscopy. Visible and near infrared range (400–2400 nm) reflectivity spectra of the GeSn optical coatings were measured using a Vis/NIR Spectrophotometer (Lambda750) at room temperature. Far-infrared range (4–18 μm) reflection spectra were measured using Fourier transform infrared spectroscopy (FTIR) with a Bruker-Tensor 27.

## Additional Information

**How to cite this article**: Jin, L. *et al*. Large area Germanium Tin nanometer optical film coatings on highly flexible aluminum substrates. *Sci. Rep.* **6**, 34030; doi: 10.1038/srep34030 (2016).
